# Mesenchymal stem cells and natural killer cells interaction mechanisms and potential clinical applications

**DOI:** 10.1186/s13287-022-02777-4

**Published:** 2022-03-07

**Authors:** Batol Abbasi, Karim Shamsasenjan, Majid Ahmadi, Seyedeh Ameneh Beheshti, Mahshid Saleh

**Affiliations:** 1 Hematology and Oncology Research Center, Tabriz University of Medical Sciences, Tabriz, Iran; 2 Student Research Committee, Tabriz University of Medical Sciences, Tabriz, Iran; 3 Stem Cell and Regenerative Medicine Institute, Tabriz University of Medical Sciences, Tabriz, Iran; 4 Stem Cell Research Center, Tabriz University of Medical Sciences, Tabriz, Iran; 5 Department of Applied Cell Sciences, School of Advanced Technologies in Medicine, Tehran University of Medical Sciences, Tehran, Iran

**Keywords:** Natural killer cell, Mesenchymal stem cells, Immunomodulation, Signaling, Therapeutic

## Abstract

Natural killer cells (NK cells) are innate immune cells that are activated to fight tumor cells and virus-infected cells. NK cells also play an important role in the graft versus leukemia response. However, they can over-develop inflammatory reactions by secreting inflammatory cytokines and increasing Th1 differentiation, eventually leading to tissue damage. Today, researchers have attributed some autoimmune diseases and GVHD to NK cells. On the other hand, it has been shown that mesenchymal stem cells (MSCs) can modulate the activity of NK cells, while some researchers have shown that NK cells can cause MSCs to lysis. Therefore, we considered it is necessary to investigate the effect of these two cells and their signaling pathway in contact with each other, also their clinical applications.

## Introduction

Natural killer (NK) cells are a member of the body's innate immune system, which have anti-tumor and anti-viral roles [1]. NK cells in leukemia patients who are candidates for hematopoietic stem cell transplantation (HSCT) play an important role in the graft-versus-leukemia (GVL) response [2]. Following HSCT, NK cells are the first population of lymphocytes that recover after HSCT, which helps improve transplantation, reduce rates of leukemia relapse, and reduce GVHD [3, 4]. However, some studies have shown that transplanted NK cells may lead to GVHD by producing pro-inflammatory cytokines (IFN-γ, TNF-a) that directly cause cell damage or indirectly by increasing the activity of transplanted T cells [3].

Mesenchymal stem cells (MSCs) are multipotent and non-hematopoietic stem cells that can differentiate into mesenchymal and non-mesenchymal tissues [5]. There are many reports that MSCs can affect the immune system by interacting with myeloid and lymphoid cells [6]. It has recently been shown that they can inhibit activity B cells [7–9], T cells [8–11], NK cells [12], dendritic cells [13, 14], and macrophages [9] through direct cell-to-cell interaction and secretion of soluble factors including prostaglandin E2 (PGE2), indoleamine‐2,3‐dioxygenase (IDO), and transforming growth factor‐beta (TGF‐β) [15–18]. MSCs due to regulate the immune system can reduce GVHD in allogeneic HSCT [19, 20]. They can escape the immune system by the lack or low expression levels of stimulatory molecules [21], and also they can survive for a long time in an allogeneic environment [22]. Therefore, their allogeneic cell products can be used for therapeutic applications to regulate the immune system in autoimmune diseases, transplantation, and tissue regeneration [23].

This review investigates how MSC could affect NK cell phenotype, proliferation, and activity. We also show signaling two cells in contact with each other and therapeutic applications of MSC on NK cell-related diseases.

## Characterization of MSCs

MSCs were identified over 50 years ago as a group of human and mammalian bone marrow cells that can be extracted and proliferated in vitro [24]. MSCs is obtained from various sources, including adipose tissue, bone marrow, skin [25], umbilical cord or peripheral blood, dental pulp, amniotic fluid, synovium, endometrium, dental pulp, heart [26], brain [27], umbilical cord tissue, and almost any graft tissue after delivery [28–31]. These cells have shown fibroblast-like appearance [32]. MSCs also characterized by other properties such as (a) adherence to plastic in culture; (b) differentiation into adipocytes, chondrocytes, and osteoblasts, under standard in vitro tissue culture-differentiating conditions; (c) expression of surface markers CD73, CD90, and CD105, and lack the expression of CD14, CD45, CD34, CD11b, CD19, CD79α, and HLA-DR in cells surface [31, 33–35]. It has shown that, depending on the tissue type, the panel of antigens may be different [6]. Table 1 shows cell surface markers of mesenchymal cells derived from different tissues [36, 37]. In the bone marrow, MSCs are essential in the development and maintenance of hematopoiesis by supplying many soluble factors such as growth factors and cytokines besides. MSCs directly interact with neighboring cells such as HSCs and extracellular matrix through adhesion molecules and extracellular matrix proteins such as integrins, ICAMs, and selectins [6].

**Table 1:** Mesenchymal stem cells markers from various tissues

	BM-MSC	AT-MSC	FB-MSC	PB-MSC	CB-MSC	PLA-MSC
Positive	CD9, CD13, CD29, CD44, CD51, CD58, CD59, CD61, CD62L, CD73, CD90, CD104, CD105, CD106, CD119, CD120a, CD120b, CD121a, CD124, CD126, CD127, CD140a, CD140b, CD146, CD166, CD271, CD340, CD349	CD44, CD90, CD105, CD106, CD146, CD166, CD9, CD13, CD29, CD54, CD73, HLA I, STRO-1	CD29, CD44, CD73, CD105	CD90, CD105, CD133	CD13, CD29, CD44, CD73, CD90, CD95, CD105, CD106, CD133, CD166	CD9, CD13, CD44, CD63, CD73, CD90, CD105, CD271, CD349
Negative	CD1a, CD3, CD4, CD5, CD11a, CD14, CD15, CD18, CD25, CD31, CD33, CD34, CD38, CD45, CD50, CD56, CD62E, CD62P, CD71, CD123, CD133, CD144, CD178	CD31, CD45, CD11b, CD14, CD19, CD34, CD79, CD133, CD144, HLA-DR	CD14, CD34, CD45, CD68	CD14, CD34, CD45	CD14, CD34, CD38, CD45, CD71	CD31, CD34, CD45

*BM* Bone marrow, *AT* adipose tissue, *FB* fetal blood, *PB* peripheral blood, *CB* cord blood, *PLA* placenta

MSCs modulate the immune system. They also have some properties of repairing damaged tissue [38]. MSCs are moved to the various tissues by chemokines released from damaged tissue and begun tissue regeneration by differentiating into organ-specific mature cells. They move from the bloodstream to damaged tissue by rolling on the endothelium, adhesion, trans-endothelial migration, extravasation [6]. Recent findings indicate that the pericyte population in the vessel might be a source of MSCs [39, 40]. Pericytes are cells that surround endothelial cells in capillaries and small plates in different organs and are essential in stabilizing blood vessels. The observation that led them to this conclusion is that cells with MSC markers also express pericyte markers. This relationship is further demonstrated by cell sorting for pericytes (CD34−, CD56−, CD45−, CD146+) and subsequent expanded into cells of multiple lineages in vitro (chondrogenic, osteogenic, adipogenic, and myogenic). Also, the expression of MSC markers by pericytes indicates that pericytes may be able to form various lines as a reservoir of precursor cells [40, 41]. In recent studies, adventitial cells (the cells that cover the outermost layer of all blood vessels except capillaries) are considered as MSCs precursors. Therefore, it can be concluded that both pericyte and adventitial cells can be precursors to MSC [42].

## Characterization of NK cell

NK cells are present in many parts of the body, including the spleen, thymus, lymph nodes, bone marrow (BM), peripheral blood (PB), uterus, and liver [43]. They can detect dying tumor cells and virus-infected cells without prior activation. They can also distinguish between normal and transformed cells by major histocompatibility complex (MHC) class I molecule expressed on cells. Normal cells express MHC class I molecule. They can engage NK cell inhibitory receptors and are protected from NK cell-mediated lysis, while malignantly transformed cells via losing the MHC class I molecules activate NK activator receptors [44]. Besides, NK cells can improve the immune system's response to target cells by increase downstream immune responses and increasing both the quality and the strength of adaptive immune defenses [45, 46]. Cell-surface NK receptors (NKRs), which recognize MHC class I and class I-like molecules signal, inhibit, and activate NK cell function, thereby enabling NK cells to identify and selectively target foreign cells [47, 48]. There are three significant superfamilies of NKRs in humans: (1) the killer cell Ig-like receptor (KIR), which primarily recognizes MHC class I molecules, (2) Ig-like transcripts (ILTs), (3) CD94–NKG2A heterodimer. These receptors are inhibitory [49]. In humans, subsets of NK cells express CD16 (FcγRIII), and also express CD56 [neural cell adhesion molecule (NCAM) or Leu-19] in high level [50]. The researchers divided human NK cells into two subsets, CD56bright and CD56dim NK cells, each with distinct phenotypes and different roles in immune responses. Approximately 90% of human NK cells are composed of CD56dim CD16bright NK cells, which are responsible for antibody-dependent cellular cytotoxicity (ADCC) through the high expression of CD16 and low expression of CD56. In comparison, about 10% of peripheral blood NK cells are CD56bright CD16dim/neg cells. Therefore, it can be said that CD56dim cells are more cytotoxic than CD56bright cells [49, 51]. Recent studies have shown that CD56bright NK cells produce high cytokines, especially Interferon gamma (IFN-γ), while CD56dim NK cells produce low levels of cytokines. Each of these subsets has a different expression of NKRs, home molecules, and cytokine receptors. For example, CD56bright NK cells express low levels of killer immunoglobulin-like receptors (KIRs) but high levels of the inhibitory CD94/NKG2A NKR. They also express the high-affinity interleukin-2 (IL-2) receptor (IL-2R), which enables them to respond to low doses of IL-2 and produce IFN-γ; and also express homing molecules CXCR3, l-selectin, and CCR7. By contrast, the CD56dim NK indicates a wide range of inhibitory NKRs, including KIR, and has low-affinity IL-2R, lacks CCR7, and low expression of l-selectin. Since CD56bright NK cells express high levels of IL-2R and T cells and DCs are the only cells that produce IL-2, it suggests these cells have cytokine interactions in vivo [51].

## MSC effect on NK cells

### Effect on the proliferation of NK cells

Several studies have shown that MSCs inhibit the proliferation of activated NK cells. In these studies, researchers investigated the inhibitory effect of MSC on NK cell proliferation with various sources of MSC, including, bone marrow-derived mesenchymal stem cells (BM-MSCs) [52, 53], adipose tissue-derived mesenchymal stem cells (AT-MSCs) [54], Wharton’s Jelly-derived mesenchymal stem cells (WJ-MSCs) [55], foreskin-derived mesenchymal stem cells (FSK-MSCs) [56]. NK cell proliferation is differentially regulated and modulated by MSCs depending on NK cell-activating cytokines. When NK cells stimulated with IL-2 [53–57], IL-12 [53–56], IL-15 [52–57], IL-21 [53–56], the presence of MSCs significantly but different extents inhibited NK cell proliferation. Spaggiari et al. [57] investigated the effect of BM-MSC on inactivated and IL-15-activated NK cells proliferation. They showed that BM-MSCs inhibited the proliferation of resting NK cells and occurred at all NK/MSC ratios tested. While BM-MSCs only partial inhibition on the proliferation of IL-15-activated NK cells and are dependent on the NK/MSC ratio. Pradier et al. [52] also showed that the proliferation of IL-15-activated NK cells is strongly inhibited by BM-MSCs. They attributed the inhibition of NK cell proliferation to BM-MSC-secreted IDO. Najar et al. [53] showed the proliferation of NK cells activated with IL-12- and IL-21 was significantly reduced by BM-MSCs, while the proliferation of IL-2 and IL-15-activated NK cells slightly decreases in the presence of BM-MSCs. They observed a slight decrease in the proliferation of IL-2-, IL-12-, IL-15- and IL-21-activated NK cells in the presence of AT-MSCs [54]. They also showed that WJ-MSCs and FSK-MSCs were significantly inhibited NK cell proliferation activated with IL-2, IL-12, IL-15, and IL-21 [55, 56]. Panagiota et al. [58] showed that factors secreted by BM-MSCs suppress NK cell proliferation, as reported for T cells. Gieseke et al. [59] investigated the effect of MSC-derived galectin-1 on the proliferation of immune effector cells. Galectin-1 is a protein that is present intracellularly and on the cell surface of MSCs. This protein is also secreted by MSCs. They have shown that BM-MSC-derived galectin-1 inhibits CD4+ and CD8+ T cells proliferation. They also investigated the effect of MSC-derived galactin-1 on the suppression of NK cell proliferation. For this purpose, NK cells were isolated using MACS technology, then stimulated with IL-2, and eventually cultured with BM-MSCs of knockdown galectin-1. They observed that galectin-1 knockdown in MSCs had no effect on inhibition of NK cell proliferation by MSC.

### Effect on apoptosis rate of NK cells

MSCs induce apoptosis into allogeneic NK cells. To determine the induction of apoptosis by MSC, Li et al. [60] exposed NK cells to BM-MSC cells in both transwell and mixed co-culture methods. They examined the cell cycle distribution of NK cells cultured with BM-MSC using flow cytometry analysis. In this analysis, the DNA fragment analysis reflects endonuclease activity in the process of apoptosis. Cells in the pre-G 0/G 1 stage (M4) were designated as apoptotic cells. They observed that in both the transwell and mixed co-culture groups, the percentage of cells in the G0/G1 phase was higher than that in the control group, whereas the rate of cells in S and G2/M phases was lower. They showed that MSC inhibits the NK cell cycle in the G0/G1 phase. The results showed that the apoptosis rate of NK cells significantly increased in both transwell and mixed co-culture groups. Besides, they established the rate of NK cell apoptosis in transwell groups was considerably higher than mixed co-culture groups. These results indicate that MSCs can affect NK cell apoptosis through soluble factors.

### Effect on cytokine production of NK cells

NK cells produce a variety of inflammatory cytokines, including IFN-γ, after active by activating receptors [50]. NK cells directly induce anti-tumor activity by secreting pro-inflammatory cytokines and chemokines such as IFN-γ, tumor necrosis factor alpha (TNF-α), interleukin-6 (IL-6), granulocyte–macrophage colony-stimulating factor (GM-CSF), and chemokine (C–C motif) ligand 5 (CCL5) and also promote innate and adaptive responses [61]. Many articles have discussed the effect of MSCs on the rate of cytokine secretion by NK cells. In most of these studies, to investigate this effect, NK cells cultured with MSC cells were considered as a positive control, and NK cells cultured in the absence of MSC as a negative control. In these trials, different results were obtained. Some studies point to increased production and secretion of inflammatory cytokines in NK cells co-cultured with MSC, but others show a decrease in the inflammatory cytokines rate. Grazia Maria Spaggiari et al. analyzed whether MSC induces IFN-γ production in NK cells, incubate NK cells at different NK/MSC ratios of autologous or allogeneic BM-MSC in the presence of the monensin-containing GolgiStop. They found that NK cells produced IFN-γ in the presence of MSC [57]. DelaRosa et al. [62] compared IFN-γ production in NK cells in their co-cultures with two hASCs and hBM-MSCs separately. They observed an increase in IFN-γ production when they used hASCs or hBM-MSCs as target cells. But there was no significant difference between hASCs and hBM-MSCs. Hu et al. [63] investigated the production of IFN-γ by NK cells after direct and indirect co‐culture with BM-MSC. They observed that IFN-γ secretion was increased (over three folds) in the co‐culture directly but was not affected by the transwell method, indicating that a contact‐dependent mechanism is involved.

IL-2-activated NK cells constitutively produced much more IFN-γ than TNF-α. Upon co-culture with AT-MSCs, the levels of both IFN-γ and TNF-α increased. The level of IFN-γ produced by IL-2-activated NK cells considerably increased, whereas the level of TNF-α was slightly raised [54–56]. In this experiment, they used different sources of MSC, FSK-MSCs [56], WJ-MSCs [55], AT-MSCs [54], and BM-MSCs [53] for co-culture with NK cell. They all observed a similar effect on cytokine production and secretion by IL-2-activated NK cells. Several other articles have seen a decrease in the production and secretion of IFN-γ and TNF-α. Debanjana Chatterjee et al. cultured IL-18 and IL-12 pre-stimulated NK cells with or without human umbilical cord derived- MSC (UC-MSC). They find out that IFN-γ production in NK cells co-cultured with UC-MSC cells compared to cultured NK cells alone decrease considerably. These findings indicate that UC-MSCs cause NK cells to respond less to stimulations by IL-12 or IL-18 or the combination of the two. They attributed this reduction in NK cells' response to IL-12 and IL-18 to a decrease in signaling IL-12R and IL-18R receptors. They stated that UC-MSCs in co-cultured with NK cells had no effect on the expression rate of IL-12R and IL-18R receptors, while they decreased signaling of these receptors by a decrease in phosphorylation pSTAT4 at receptor IL-12R receptor and NF-kB at IL-18R receptor. Moreover, they also attributed the downregulation of IFN-γ production in NK cells to activin-A produced by UC-MSCs by reducing T-bet levels in NK cells. A T-bet transcription factor is a significant modulator of IFN-γ production in NK cells and T cells. The addition of Activin-A to the NK cell culture medium could substantially decrease IFN-γ production level in response to IL-12/IL-18. On the other hand, MSCs produce Activin-A constructively that Activin-A secretion increased from MSCs when exposed to NK [64].

In addition to affecting the production and secretion of IFN-γ and TNF-α [65], MSCs also effect on IL-10 and IL-6. Liu et al. [66] showed a decreasing effect on the production and secretion of IL-6, IFN-γ, and TNF-α and an increased effect on IL-10 by NK cells co-cultured with MSCs, whereas, Ma Qingqing et al. by evaluating the impact of BM-MSC on NK cells, found that BM-MSCs reduced TNF-α and IFN-γ secretion from NK cells, while they had no effect on IL-10 [67]. Two other studies have also shown that BM-MSCs and AT-MSCs suppress the production and secretion of IL-10 from NK cells [58, 68].

### Effect on degranulation of NK cells

The NK cells cytolytic function is accomplished through various processes death receptor binding or degranulation. They are vital for the scavenging of diseased and disordered cells [50]. CD107a is a marker of T-cell CD8+ and NK cell degranulation [69]. Since these membrane proteins appear on the cell surface after exocytosis of cytotoxic granules (CGs), it is hypothesized that some of these proteins may involve in the temporarily keeping of cytotoxic lymphocytes from self-degraded [70]. Several studies have reported the effect of MSC on NK cells degranulation by measuring CD107a expression during 5 days of NK cell co-cultured with MSC. They observed that the percentage of CD107a-expressing NK cells continuously increased until day 5, but this increase was sustained in the presence of MSCs during period continuously and time-dependent. In this experiment, they used different sources of MSC, FSK-MSCs [56], WJ-MSCs [55], AT-MSCs [54], and BM-MSCs [53] for co-culture with NK cell. They all observed a similar effect on NK cells degranulation, whereas DelaRosa et al. [62] compared the sensitivity of hASCs and hBM-MSCs to NK cell-mediated lysis by degranulation assays by finding CD107a/b at the surface molecule. Since the degranulation is associated with NK cell cytotoxicity, first NK cells stimulated by rhIL-2 to increase their cytotoxicity, then both hASCs and hBM-MSCs cells used as target cells. The results showed that the degranulation rate in response to hASCs was meager and not statistically significant. In contrast, hBM-MSCs induced a significantly increase in the NK cell degranulation response. To exclude the inhibitory effect of HLA class I, they blocked HLA class I in target cells by pretreating target cells with monoclonal antibody W6/32 before co-culture. The results showed that HLA class I coated by antibody did not increase the degranulation of NK cells against hASCs. Bruno et al. [71] did the same experiment with this difference that NK cells were stimulated by IL-15 rather than by rhIL-2 to increase their cytotoxicity potential and also used human liver stem cells (HLSCs) and BM-MSCs cells as target cells. They observe MSCs induced degranulation in NK cells, whereas degranulation is absent in NK cells in response to HLSCs.

### Effect on cytotoxicity of NK cells

Cytotoxic T lymphocytes (CTLs) and natural killer (NK) cells are effective lymphocytes that use common cytotoxic pathways to defend against cancer cells and virus-infected cells. Both types of cells destroy their cellular targets using both mechanisms that require direct contact between the effective and the target cells [72]. In the first pathway, they release the contents of their granules, including perforin (responsible for the formation of pores in the cell membranes of the target cells) [62] and of the granzymes (family of serine proteases that do a series of proteolytic events) [70] and lead to the death of the target cell. The second pathway involves the involvement and accumulation of target-cell death receptors like FAS (CD95), by their homogeneous ligands, such as FASL ligands (FASL), on the killer-cell membrane, that leads to classical caspase-dependent apoptosis [73]. This pathway destroys self-reactive lymphoid cells [74]. Several studies have shown that IL-2-activated NK cells constitutively release significant perforin and granzyme that granzyme A greater secretion rate than granzyme B. They stated that the secretion rate of both perforin and granzyme increased in the presence of MSCs [54–56]. In this experiment, they used different sources of MSC, FSK-MSCs [56], WJ-MSCs [55], AT-MSCs [54], and BM-MSCs [53] for co-culture with NK cell. They all observed a similar effect on the cytotoxic activity of NK cells, while Fu et al. showed that NK cell perforin secretion decreased after co-culture with decidua MSC (DMSC) [75]. Also many studies have shown that the cytotoxicity of NK cells suppressed by MSCs [23, 32, 52, 63, 68, 76]. Chatterjee et al. [4] stated that UC-MSCs reduce the cytotoxic activity of NK cells through a decrease in the expression of NK cell surface receptors that are essential for their activity, not by altering the contents of the perforin and granzyme.

### Effect on expression receptors of NK cell

NK cells have two groups of receptors called inhibitory and activating receptors on their surface that their activity controlled by these receptors [77]. These receptors have their ligands that can activate or inhibit NK cells [78]. Table 2 summarizes these activation and inhibitory receptors and other markers [79–82].

**Table 2:** Human NK cell markers and receptors

	Ligands
*Activating receptors*	
CD94/NKG2C	HLA-E
KIR2DL4 (CD158d)	HLA-G, HS
KIR2DS1 (CD158h)	HLA-C
KIR2DS2/3 (CD158j)	HLA-C
KIR2DS4 (*CD158i)*	HLA-A, C
KIR2DS5 (CD158f)	????
KIR3DS1 (CD158e2)	HLA-B
2B4 (CD244)	CD48
FCɣRIII (CD16)	IgG
NKp46 (335)	Viral antigens
NKp44 (336)	Viral antigens
NKp30 (337)	B7-H6
NKG2D (CD314)	MIC-A, MIC-B, ULBPs
DNAM-1 (CD226)	Nectin-2 (CD112) PVR (CD155)
NKp80	AICL (activation-induced C-type lectin)
NKRP-1 (CD161)	LLT-1
*Inhibitory receptors*	
NKG2A/CD94	HLA-E
KIR2DL1 (CD158a)	HLA-C2
KIR2DL2/3 (CD158b)	HLA-C1
KIR2DL4 (CD158d)	HLA-G
KIR2DL5 (CD158f)	????
KIR3DL1 (CD158e1)	HLA-B
KIR3DL2 (CD158k)	HLA-A
LIR-1	HLA-A, -G
TIM-3	GAL-9
CEACAM1	CEACAM1/5
PD1 (CD279)	PD1L(CD274 or B7-H1)
KLRG-1	Cadherins
TIGIT	PVR, PVRL2
*Death receptors and ligands*	Ligands
TRAIL (TNF-related apoptosis-inducing ligand or CD253)	DR4 (TRAIL-R1), DR5(TRAIL-R2)
Fas ligand (CD95L)	CD95
Fas or Apo1 (CD95)	CD95L
CD40L (CD154)	CD40
*Other antigens*	
TNFRSF7 (CD27)	CD70
LAMP-1 (CD107a)	–

#### Activating receptors

When NK cells are activated, the expression of activating receptors on their surface increases, but in the presence of MSC, the expression of these receptors decreases [76, 83, 84]. In several studies, NK cells stimulated by IL-2, IL-12, IL-15, and IL-21 and measured the number of CD226 (DNAM-1), CD314 (NKG2D), CD335 (NKp46), CD336 (NKp44), and CD337 (NKp30) expressed on their surface. They observed that after the activation of NK cells by these cytokines, the rate of expression of activating receptors on their level increased, but in co-culture with MSC cells, the quality of expression of activating receptors decreased [53–56]. In this experiment, they used different sources of FSK-MSCs [56], WJ-MSCs [55], AT-MSCs [54], and BM-MSCs [53] for co-culture with NK cell. They all observed a similar effect on the expression of activating receptors NK cells. Spaggiari et al. [85] examined the rate of NKp30, NKG2D, NKp44, NKp46, CD69, 2B4 (CD244), IL-2R-8 chain (CD132), and CD16 expression on the surface of NK cells after activation with IL-2 and after co-culture with BM-MSC. They showed that the expression of activating receptors, including NKp30 and NKG2D, on NK cells increased after activation with IL-2, and NKp44 receptor and the activation marker CD69 expressed, while they are not present in inactivated cells. After co-culture with BM-MSC, NKp30, NKp44, and NKG2D expression decreased, and surface density of NKp46 and CD69 no reduced, and surface expression of 2B4 and CD132 inhibited, but it did not affect CD16 molecules. While Petri et al. [86] stated that when human nasal mucosa-derived MSCs (nm-MSCs) stimulated by poly (I:C), they secrete TGF-β and IL-6, which in co-culture nm-MSC with NK cell, these two compounds reduce the expression of CD16, and also Abumaree et al. [84] stated that in IL-2-activated NK cells and cultured with placental MSCs (pMSC) for 24 h, NKp46 and, CD69 expression were reduced.

#### Inhibitory receptors

When NK cells activated, inhibitory receptors on their surface reduced, while their expression increased in co-culture with MSC. Yan et al. [76] stated that CD158a and CD158b inhibitory receptors on NK cells are increased by co-culture with human bone marrow mesenchymal stem cells (BMMSCs) and dental pulp stem cells (DPSCs), while Spaggiari et al. [85] showed in co-culture of IL-2-activated NK cells with BM-MSC no changes were observed in the expression KIRs and CD94/NKG2A receptors, and also Abumaree et al. [84] observed a decrease in CD94 expression on IL-2-activated NK cells after co-culture with pMSC.

### Effect on expression other markers of NK cell

There are many markers on the surface of NK cells. They play an essential role in NK cell activity. In the interaction of NK cells with MSC, MSC changes these markers' expression on the surface of NK cells. Yan et al. [76] stated that the percentage of CD73 NK cells in peripheral blood is low, but in the interaction of NK cells with BMMSCs and DPSCs, the expression of CD73 on NK cells increases that these CD73‐positive NK cells can regulate the NK cells function in autocrine and paracrine process. Previous studies have shown that NK cells have CD25 (IL-2R) and CD69 activation receptors on the self-surface that proliferation potential is shown by CD25. At the same time, CD69 is a specific marker for the cytotoxic activity of NK cells [87]. Li et al. [60] stated that the expression of CD69 in NK cells decreases after co-culture with BM-MSC that this reduction is more evident at higher MSC concentrations the higher. This decrease in CD69 expression can be a factor in reducing the cytotoxic activity of NK cells. MSC also affects the expression of other markers on the surface of the NK cell. For example, in NK cells cultured with pMSC in (25:1, 50:1, and 100:1) ratios after 24 h, IL-18Rβ expression increased in all ratios and IL-12Rβ1, IFN-ɣ R1, and TLR3 expression increased in the ratio 25:1, while IL-12 Rβ1 expression decreased in the ratios 50:1 and 100:1 and IFN-ɣ R2 expression decreased in all ratios, but there was no change in the expression IL-18 Rα, TNF-α, TLR7, and TLR9 [84]. Petri et al. [86] also showed that when nm-MSCs stimulated by poly(I:C) and secretes TGF-β and IL-6, cause increases CXCR4 expression on NK cell. They attributed the increase in expression of CXCR4 on NK cells to TGF-β and stated that IL-6 has an increasing effect on TGF-β function.

### Effect on the phenotype of NK cell

As mentioned earlier, one of the markers present on the NK cell surface and affects its phenotype is CD56. Based on the expression rate of CD56, NK cells are divided into two categories, CD56dim, and CD56bright. When NK cells are stimulated with IL-15 or IL-2 in vitro, CD56bright cells have a higher proliferation than CD56dim cells. In contrast, CD56dim cells have a relatively low expansion, so after 4 days they are exposed to IL-15 or IL-2, NK-cell cultures consist mainly of CD56bright cells [58, 88]. Sotiropoulou et al. [58] cultured NK cells with BM-MSC in both contact and transwell systems. They observed that CD56 expression was high in transwell systems as seen in NK cells in the absence of MSC, while decreased in the contact system. Also, the percentage of CD56bright cells decreased in the presence of MSC that was mainly contact-dependent [58, 68, 88]. They also stated that CD56 expression levels were lower in the CD56bright cells cultured contact with BM-MSCs between groups, while in CD56dim cells, it was unchanged [58].

## NK cell effect on MSCs

### Effect on the expression of MSC ligands

To investigate the interactions between NK cells and MSCs, the researchers decided to analyze ligands expression of MSC surface recognized by NK cell-activating or inhibitory receptors. They identified surface ligands of MSCs using specific mAbs. Spaggiari et al. [57] showed the expression of MICA and ULBPs (both ligands of NKG2D), Nectin-2 and PVR (both ligands of DNAM-1), and low levels of HLA class I molecules among 15 populations of BM-MSC. However, none of the BM-MSCs were expressed CD48 (ligand 2B4) molecule. M. H. Abumaree et al. analyzed the expression rate of ligands of pMSCs and decidua parietalis derived-MSCs (DPMSCs) surface related to NK cell-activating and inhibitory receptors by flow cytometry. They stated that the highest expression at the pMSCs surface, was ULBP-2 and the lowest expression was PVR. They also showed that 95.60 percentage of pMSCs expressed HLA-ABC and 30.93 percentage expressed HLA-E [84]. They also stated that DPMSCs expressed high levels of Nectin-2 and HLA-ABC ligands and expressed low levels of MICA, MICB, ULBP-2, and HLA-E ligands [89]. DelaRosa et al. [62] compared ​ligands related to NK cell-activating or inhibitory receptors on the surface of both hBM-MSCs and hASCs. They showed that the expression of the HLA class I molecule and Nectin-2 and PVR ligands were higher in hBM-MSCs than hASCs, and the CD48 molecule was not expressed in both cells. They also stated that the expression of ULBPs and MICA / B ligands in hASC is negative or very low. They then examined the expression rate of ligands in inflammatory conditions. For this purpose, hASCs and hBM-MSCs were stimulated with IFN-γ for 72 h, and then the expression of ligands was evaluated. They showed expression of CD48, MICA/B, Nectin-2, and PVR did not increase significantly, while the expression of HLA class I and II molecules increased after 72 h.

Mehdi Najar et al. investigated the expression rate of MSC ligands from various tissues after co-culture with un-activated and activated NK cells. For this purpose, NK cells are first activated by IL-2, IL-12, IL-15, and IL-21 and then co-culture with MSCs. They stated that in BM-MSCs, the expression of Nectin-2 and PVR did not show any significant change after co-culture with inactivated or IL-21-activated NK cells, while in the presence of IL-12-activated NK cells showed a slight decrease and in the presence of IL-2- and IL-15-activated NK cells showed a more significant reduction [53]. In the case of AT-MSCs, the expression of Nectin-2 and PVR did not show any significant change against IL-12- and IL-21-activated NK cells, while showed a striking reduction in the presence of IL-2- or IL-15-activated NK cells [54]. They also stated that the expression of these two ligands remained unchanged at WJ-MSCs surface in the co-culture with inactivated and IL-21- or IL-12-activated NK cells, but it had a slight but significant reduction in co-culture with IL-15 or IL-2-activated NK cells [55]. The expression rate of Nectin-2 and PVR ligands on the FSK-MSCs surface after co-culture with un-activated, IL-12- or IL-21-activated NK cells were slightly affected. While a minor decrease in these ligands expression by FSK-MSCs was seen in the presence of IL-2-activated NK cells, a significant reduction was seen in the presence of IL-15-activated NK cells [56]. No change in ULBP3 expression was observed in all tests and in all MSC sources [53–56].

### Effect on MSC lysis rate

Many studies have shown that MSCs were lysed by activated NK cells, not freshly isolated. Mehdi Najar et al. showed that activated PBMCs by PHA/IL-2 killed MSCs isolated from different tissues (BM-MSCs, AT-MSCs, WJ‑MSCs, and FSK-MSCs). To identify the cell subpopulation involved in the death of MSCs, MSCs from various tissues were cultured with PBMCs lacking CD14, CD56, or CD3 cell population separately. They showed that activated PBMCs and without CD3+ or CD14+ cell population could not affect MSCs viability. Therefore, it shows that neither of these two immune cells is responsible for the death of MSCs, while activated PBMCs without CD56+ cell populations were not able to kill MSCs. They concluded that NK cells are the leading cause of MSC death. They then showed that inactivated NK cells did not kill MSCs, while MSCs are lysed in the presence of activated NK cells with IL-2-, IL-12-, IL-15- or IL-21. In addition, they stated that the percentage of lysed MSCs by activated NK cells varies depending on the activating cytokine type [53–56]. Also, Panagiota et al. [58] investigated the effect of inactivated and IL-15-activated NK cells on BM-MSC, and Hoogduijn et al. [90] examined the effect of inactivated and L-2- and IL-15-activated NK cells on AT-MSC, and Spaggiari et al. [57] investigated the effect of inactivated and IL-12- activated NK cells on BM-MSC, and Abumaree et al. [84, 89] examined the effect of inactivated and IL-12-activated NK cells on pMSC and DPMSC. They also stated that inactivated NK cells could not lysis MSCs, while cytokine-activated NK cells killed MSCs. The death rate of MSCs by activated NK cells is dependent on the dose of NK cells and has increased with the increasing number of NK cells compared to MSC [90, 91]. The researchers examined whether there was a difference in the lysis of allogeneic and autologous MSCs. They found no difference between autologous and allogeneic MSCs in lysis by NK cells [57, 90].

To determine which receptor-ligand interactions causes lysing MSCs by NK cells, the researchers used the mAb-mediated blocking method in cytotoxicity assays. Spaggiari et al. [57] stated that mAb-mediated blocking NKG2D, NKp30, and DNAM-1 on BM-MSC surface resulted in the inhibition of kill. But the blocking of NKp46 resulted in low inhibition. However the masking of NKp44 did not play an important role. They also showed that BM-MSCs lysis did not increase with blocking the HLA class I and the CD94/NKG2A (HLA-E specific inhibitory receptor). Abumaree et al. [84] stated that mAb-mediated blocking NKp46, NKp44, NKG2D, NKp30, and DNAM did not inhibit the lysis of pMSCs by NK cells. They also showed that blocking CD94/NKG2A did not affect killing pMSC by NK cells, while mAb-mediated blockade of the activating receptor CD69 leads to inhibition of pMSCs lysis by NK cells. Abumaree et al. [89] performed the same test on the DPMSCs. They showed antibody-mediated masking of NKG2D, NKp30, and NKp44 significantly inhibited DPMSCs lysis by NK cells, while the blocking CD69 leads to moderate inhibition of NK cell-mediated DPMSCs lysis. But the blocking NKp46 and DNAM-1 did not inhibit NK cell lysis of DPMSCs. They also showed that blocking CD94/NKG2A did not increase the killing of DPMSCs by NK cells. Several studies have attributed the increase in cytotoxicity of NK cells against MSCs to the decreased serpins expression of MSCs during co-culture with NK cells and the effect of granzyme on MSCs. As previously mentioned, NK cells exert their cytolytic activity on target cells through the secretion of granzyme. However, excessive activity of granzyme causes damage to tissue cells. MSCs can inhibit the granzyme cytotoxic effect of NK cells by expressing serpins, a family of protease inhibitors, and thus escape from lysis by NK cells [92]. Previous reports have shown that serpin B9 protects MSCs against activated NK cells-mediated granzyme-dependent killing [93]. Several studies have shown high expression of serpin B9 in MSCs isolated from various sources (WJ-MSCs [55], BM-MSCs [53], AT-MSCs [54], and FSK-MSCs [56]). On the other hand, Mangan et al. showed that serpin B9 is inactivated by reactive oxygen species (ROS) [94]. Mehdi Najar et al. investigated the expression of serpin B9 and ROS production in various sources of MSCs (WJ-MSCs, BM-MSCs, AT-MSCs, and FSK-MSCs) during co-culture with IL-2-activated NK cells. They showed increased ROS production, decreased serpin expression and increased lysis susceptibility in all MSCs during co-culture with IL-2-activated NK cells [53–56]. However, the researchers showed that MSCs were protected from lysis by NK cells by increasing IFN-γ during inflammatory responses. Spagiari et al. [57] in a study on BM-MSC, and Giuliani et al. [95] in a study on fetal-MSC (FL-MSC) and embryonic-MSC (ES-MSC), investigated the effect of IFN-γ on the protection of MSC against NK cells. They showed that IFN-γ increased HLA class I and HLA-E molecules expression in MSCs and decreased lysis of MSCs by NK cells. To confirm this hypothesis, they blocked HLA-I and CD94/NKG2A receptor by mAb. They observed that the killing of IFN-γ-treated MSCs by NK cells increased, indicating their role in MSCs protection. Hoogduijn et al. [90] examined whether immunosuppressive drugs could protect MSCs lysis against NK cells. To do this, they added tacrolimus, rapamycin, or sotrastaurin to NK cells together with MSCs. They observed that none of the immunosuppressants could prevent the lysis of allogeneic or autologous MSCs by activated NK cells. They repeated the experiment by pre-incubating activated NK cells with immunosuppressants for 24 h and also before and during NK cell activation with IL-2 and IL-15. Therefore, NK cells were incubated with rapamycin, tacrolimus, or sotrastaurin for 24 h before activation and then cultured with IL-2 and IL-15 for 7 days in the presence of the same drugs. They observed that none of the immunosuppressants could protect MSCs from lysis against NK cells.

## Mechanisms involved in inhibiting NK cell function by MSC

### Role of soluble factors in the immunoregulatory activity of MSCs

In several studies, the cytotoxic suppression of NK cells by MSCs attributed to enzymes cyclooxygenase (COX)-2 [4], and IDO [52, 63, 96] and also their metabolites, respectively, prostaglandin (PG)-E2 [4, 63, 96] and kynurenine [52, 63] produced by MSCs. They found significant concentrations of l-kynurenine induced by the degradation of l-tryptophan by IDO [52] and PGE2 end-product COX-2 [4] by PGE2 synthase [63] in the NK-activated MSC medium. They proved their theory by using antibodies blocking these enzymes and their metabolites. They also stated that initial cell contact between MSC and NK cells is required to induce the inhibitory effect through soluble factors synthesized by MSC. MSCs require preincubation with NK cells, and activation via IFN-γ is derived from these cells [97]. They first cultured BM-MSCs with IL-15-activated NK cells. They observed a significant concentration of l-kynurenine in the IFN-γ-activated MSCs medium cultured with IL-15-stimulated NK cells, and then observed l-Kynurenine synthesis was inhibited by blocking anti-IFN-γ mAb [52]. Also, Chatterjee et al. [98] stated that IL-1β produced by NK cells causes COX-2 upregulation in MSCs. They added IL-1β neutralizing antibody in NK-MSC co-cultures for confirming their hypothesis, which observed a significant inhibition of COX-2 upregulation. They also stated that the interaction between the two cells was dependent on cellular contact.

In one study, the effects of fetal liver (FL) MSC-derived exosomes (FL-exo) and MSCs derived from the human fetal liver (FL-MSC) on NK cells activity were investigated. They observed that FL-exo and FL-MSC impaired the activity of NK cells. They performed a proteome analysis by mass spectrometry to identify factors related to the inhibitory effects of exosomes. This analysis revealed the presence of TSP1, which is a latent TGFβ activator through the conformational change in the latency-associated peptide (LAP). They also showed surface expression of TSP1 on FL-exo by flow cytometry. Immunoprecipitation of LAP, a component of the latent TGFβ complex, showed the co-precipitation of TGFβ and TSP1, indicating the interaction of LAP with TGFβ and TSP1 on FL-exo. They also examined whether the exosome induces the TGFβ/Smad pathway on the NK cell. They found that IL-2-activated NK cells did not exhibit Smad2/3 phosphorylation but after treatment with TGFβ or FL-exo induced Smad2/3 phosphorylation in NK cells, which concluded that the inhibitory effect of exosomes on NK cell function is dependent on surface expression of TGFβ on FL-exo. They used an anti-TGFβ neutralizing antibody to prove their theory. They observed that NK cells' activity was restored by these antibodies, thus suggesting that the inhibitory effect of FL-exo and FL-MSC on NK cells activity partially dependent on their TGFβ expression [23]. Eventually, MSCs inhibit the cytotoxic activity of NK cells by a decrease in exocytosis and decrease in perforin release by NK cells via release of these soluble factors [52].

### Role JAK/STAT cytokine signaling

NK cells need JAK/STAT signaling in response to more than 50 cytokines, hormones and growth factors to the regulation of differentiation, survival, proliferation, migration, and cytotoxicity [99–101]. The JAK/STAT signaling cascade can transmit extracellular signals from the cell membrane to the nucleus through various stages. When cytokines bind to the corresponding receptor, receptor-related JAK kinases come into proximity, and resulting JAKs phosphorylate each other and also the intracellular part of the receptor, and several downstream signaling molecules, including STATs. STAT phosphorylation causes them to dimmer with other STATs and separate them from the membrane complex and migrate to the nucleus [102]. In the nucleus, STATs regulate gene expression and subsequently affect cell proliferation and activity [103]. JAK family have four members (JAK1-3 and TYK2) and STAT proteins are STAT1-4, STAT5A, STAT5B, and STAT6. Although individual members of the JAK/STAT cascade have a high homologation, their specific functions are significantly different. Each cytokine can capture other JAK and STATs in the JAK-STAT signaling pathway and affect the cell [102]. For example, by binding IL-10 to its receptor complex, phosphorylation of JAK1 and TYK2 proteins occurs, and eventually, STAT3 is phosphorylated, which suppresses the immune system [104], while suppressor of cytokine signaling (SOCS) family proteins can suppress the JAK kinases activity [105] and ultimately inhibit the action of the JAK/STAT signaling cascade [106] via competing with the STAT proteins for binding to the receptor [102]. In mammals, four family members (CISH, SOCS1, SOCS2, and SOCS3) are associated with the JAK-STAT pathway and inhibit this pathway [101]. Liu et al. [66] showed in co-culture mice MSC with mice NK cells, MSC affects the properties and function of NK cells by regulating the JAK-STAT signaling pathway and SOCS3, a downstream component of the JAK/STAT signaling pathway. They stated that IL-10 regulates NK cell activity through the JAK/STAT pathway. To prove their theory, they adding AG490 as a specific inhibitor of the JAK/STAT pathway to the medium of NK cells cultured with MSC. They showed that the proliferation and expression of NK cell surface markers returned to normal levels before co-culture with MSCs.

### Role CD73 expressed on the level of NK cells in inhibiting their activity

Ecto-5′-nucleotidase (CD73) and E-NTDPase (CD39) are ectoenzymes located on the membrane surface of immune cells, including T-cells, NK cells, B-cells, dendritic cells, and macrophages [107]. Purinergic (P1 and P2) signaling pathways play an essential role in regulating the immune system [108]. Adenosine (via P1) and ATP (via P2) are usually present in small amounts in extracellular fluids [109]. However, high levels of ATP are released in the cancer microenvironment due to cell destruction [110] that this extracellular ATP functions as a danger-associated molecular pattern (DAMP) and increases the immune response to cancer cells [108, 110]. CD39 by converting ATP to AMP and CD73 by converting AMP to adenosine suppress the immune system [111]. As a result of the extracellular ADO accumulation, followed by the involvement of ADO receptors with G protein (A1, A2A, A2B, and A3), the function of immune cells, including NK cells, is suppressed through various mechanisms [112] that tumor can use this mechanism to escape the immune system [113], while the loss of function of ADO production can cause autoimmune disorders (i.e., encephalomyelitis, multiple sclerosis, rheumatoid arthritis, diabetes, and uveitis) [114]. Yan et al. [76] stated that BMMSCs and DPSCs in co-culture with NK cells increase CD73 expression on NK cells, which suppresses NK cell function.

### Effect on LAIR pathway of NK cell

The leukocyte-associated Ig-like receptor-1 (LAIR-1; CD305) is an ITIM-containing inhibitory immune receptor with a single immunoglobulin-like domain and a cytoplasmic tail, which is expressed on most blood leukocytes, including NK cells [115]. LAIR-1 is located on the surface of NK cells and activated when they bind to their ligands. They employ SHP-1 and SHP-2 phosphatases after activation, and leading to potent inhibition of NK cell toxicity [116]. One of the most essential LAIR-1-ligands is collagen [75, 117], which is abundant in the human body [118]. However leukocyte-associated Ig-like receptor-2 (LAIR-2; CD306) has homology with hLAIR-1, which can also be secreted. They lack a transmembrane and cytoplasmic area that competes with LAIR-1 to connect to the ligand [75, 119]. Therefore, the LAIR-2 secretory receptor regulates the inhibitory potential of the membrane-bound hLAIR-1 through competition for the same ligands [75]. Najar et al. [55] investigated the effect of MSC from various sources on NK cell CD305 and CD306 expression after co-culture. They showed that unactivated NK cells and IL-2-activated NK cells express CD305 and CD306 on themselves, but in the co-culture of NK cells with MSC, their expression rate changes. They observed that when NK cells were co-cultured with WJ-MSC, the expression of both CD305 and CD306 markers decreased, while in co-culture with FSK-MSC, increased the CD305 expression but, the CD306 expression remained unchanged [56]. They also showed that in the co-culture of NK cells with BM-MSC, CD305 expression remained unchanged, but CD306 expression decreased [53] and in co-culture with AT-MSC, expression of both markers remained unchanged [54]. The integrity of the triple collagen helix depends on the hydroxylation of proline by P4H. LAIR-1 possibly binds to the collagen triple helix peptides that contain several replications of glycine-proline-hydroxyproline [120]. Fu et al. [75] stated that decidua MSCs (DMSCs) can affect NK cell function by producing collagen and the interaction between collagen and LAIR-1. To prove this hypothesis, they disrupted collagen post‑transcriptional modification by transfecting DMSCs with P4H shRNA. They observed inadequate proline hydroxylation of collagen and eventually altered the ability of collagen to bind to LAIR-1.

### Mechanism of cell-to-cell contact between MSC and NK cells

**Fig. 1:**
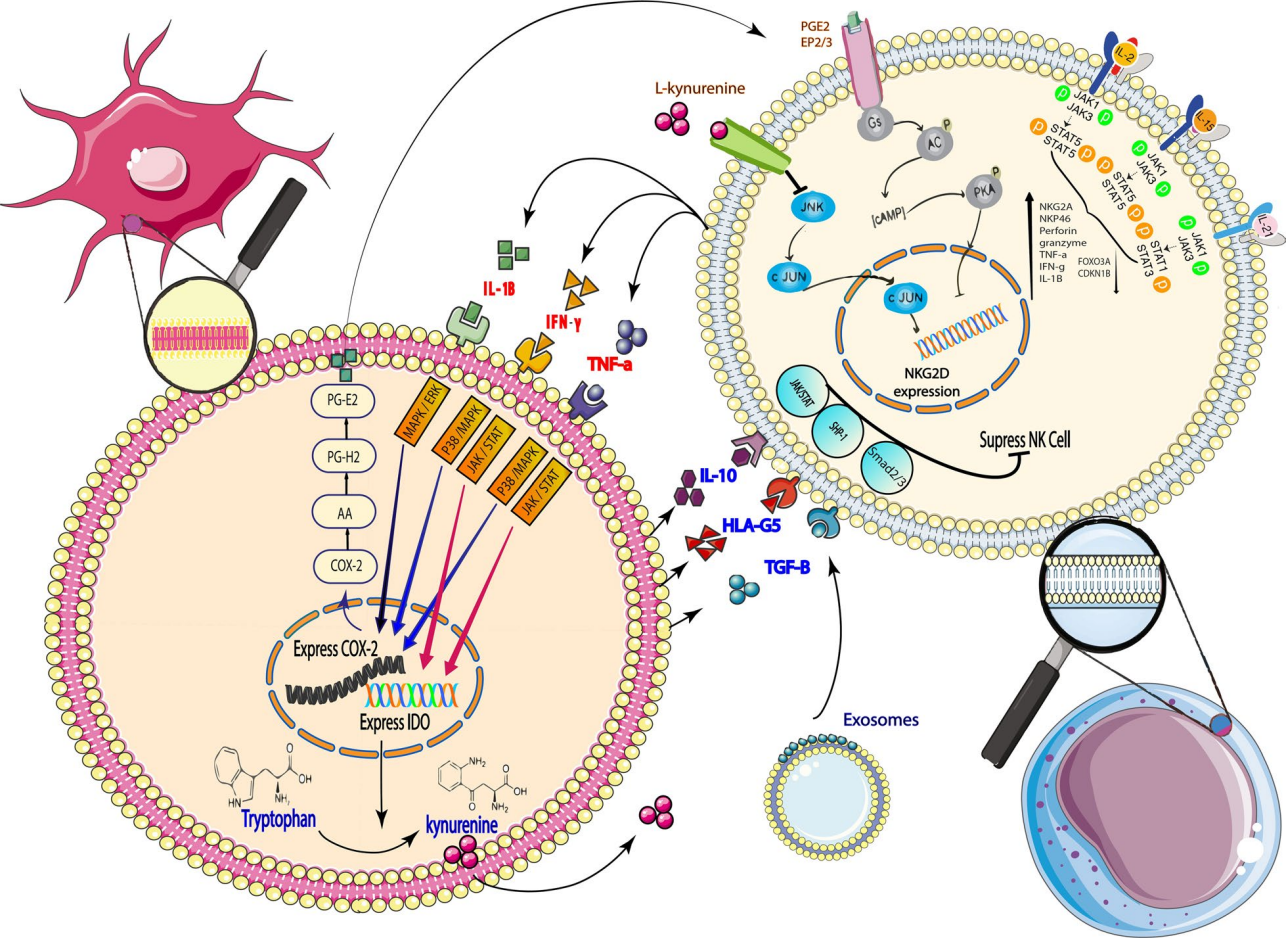
Mechanism of cell-to-cell contact between MSC and NK cells. IL-15-, IL-2-, IL-21-stimulated NK cells release inflammatory cytokines. These inflammatory cytokines affect the MSC and caused the release of PGE2 and kynurenine and other factors from the MSC. The MSC inhibits the function of NK cells by these factors. *PGE2* prostaglandin E2, *IL-15* interleukin-15, *IL-2* interleukin-2, *IL-21* interleukin-21, *COX-2* cyclooxygenase-2, *IDO* indoleamine-2,3-dioxygenase, *AA* acid arachidonic, *PG-H2* prostaglandin H2, *IL-1β* interleukin-1β, *IFN-γ* Interferon gamma, *TNF-α* Tumour Necrosis Factor alpha, *EP2/3* Prostaglandin receptor, *FOXO3A/CDKN1B* quiescence-associated genes, *IL-10* interleukin-10, *HLA-G5* Human leukocyte antigen-G5, *TGF-β* Transforming growth factor beta, *STAT4* signal transducer and activator of transcription, *JAK* Janus kinase, *NK cell* natural killer cell

IL-15-, IL-2-, IL-21-stimulated NK cells can produce inflammatory cytokines, including IFN-γ, TNF-α [102, 121], IL-1β [122] through the JAK-STAT signaling pathway. IFN-γ and TNF-α affect MSC and increase COX-2 and IDO expression [123–125], IL-1β also increases COX-2 expression [125–127]. Finally, PGE2 produced by COX-2 [128, 129], and is kynurenine produced from tryptophan by IDO [130]. PGE2 released from the MSC through EP2 and EP4 that are NK cells receptors [131–133] inhibits NKG2D expression [134, 135]. Also, activated adenylate cyclase in the PGE2 signaling pathway also increases the expression of the CD94/NKG2A inhibitory receptor on NK cells [136]. Kynurenine released by the MSC also inhibits NKG2D expression by NK cells [134, 137]. In this way, PGE2 can decrease the production of IFN-γ and TNF-α by NK cells [129, 132, 138]. MSC can also inhibit NK cell function by producing HLA-G, TGF-β, IL-10, and HGF [96, 123]. As previously mentioned, TGF-β reduces NK cell proliferation [123] through the Smad2/3 pathway [23]. HGF also inhibits the function of NK cells by suppressing IFN-γ and TNF-α expression [123]. HLA-G5 is an ILT2 inhibitory receptor-ligand on NK cells that inhibits NK cell function after binding to this receptor [139]. IL-10 also regulates NK cell function through the JAK/STAT signaling pathway [66] (Fig. 1).

## The relationship between MSC–NK interactions with the incidence and therapy of diseases

### Association of MSC–NK interactions with malignancies

As mentioned earlier, MSCs can reduce the activity of NK cells. In one study, they compared the effect of MSC on NK receptors between two groups of MSC, non-tumor tissue-derived MSCs (N-MSCs) and lung-tumor-derived MSCs (T-MSCs). They found that T-MSCs reduces the expression of activating receptors of NK cells more than N-MSC [88]. T-MSCs also alter NK cell phenotype. They can increase the ratio of NKCD56dim: NKCD56bright cells. As we know, NKCD56bright cells are better producers of IFN-γ, while NKCD56dim cells have less protection against tumors that could be one of the reasons for tumor escape from the immune system and tumor progression [140]. On the other hand, the tumor environment contains tumor stromal cells (TSC), which have functional and morphological characteristics similar to MSCs and assumed MSCs may be the source of these stromal cells [141] and harm NK cells by reducing NKp44 and NKp46 expression [142]. Cytokine-activated NK cells are injected into cancer patients after HSC transplantation to eliminate the residual tumor cells [143]. Because the BM transplant contains small amounts of MSCs that survive for a long time in the host, they can have inhibitory effects on injected NK cells [144]. While some researchers, in addition to the suppressive effect of MSC on the immune system, observed the immune system's stimulating effects. Entrena et al. [145] used cells isolated from patients with acute lymphoblastic leukemia (ALL) in low-/moderate- and high-risk individuals to evaluate the suppressive or stimulating effect of MSC on the immune system. They observed that MSC isolated from low- and moderate-risk patients increased NK cell cytotoxicity, while MSC isolated from high-risk patients suppressed the anti-tumor function of NK cells. Therefore, they concluded that the effect of MSC on the immune system depends on the specific microenvironment of the tumor. For this reason, researchers are searching for MSC cells with the stimulatory properties of NK cells.

### Association of MSC–NK interactions with autoimmune diseases

As mentioned earlier, excessive reactions of NK cells can cause a series of diseases. For example, NK cells can be a significant cause of virus-related asthma [146]. Also, in the peripheral blood of people with asthma, the activity of NK cells is increased [147], which migrates through the circulation toward lungs and lymphoid organs [147, 148]. On the other hand, NK cells promote the response to the allergen through T cells [149, 150]. Numerous studies have also shown an increase in the number and activity of NK cells in patients with multiple sclerosis (MS). They attack and damage myelin-producing oligodendrocytes [151–153]. NK cells found in the pancreas of patients with type 1 diabetes, while not seen in healthy people. Engagement of the NKP46 receptor of NK cells with pancreatic beta cells causes degranulation NK cells eventually cause disease [154]. In the synovial fluid of rheumatoid arthritis (RA) patients, an accumulation of NK cells was observed, which the researchers attributed the progression of the disease to the NK cells [155]. They also observed that patients' synovial fluid have more CD56bright NK cells secreting IFN-γ than their peripheral blood [156], while in patients with systemic lupus erythematosus (SLE), there is a change in the ratio of CD56bright: CD56dim and an increase in the CD56bright subsets [157, 158]. As mentioned previously, the cause of autoimmune diseases is an increase in the number or activity of NK cells. They can also differentiate activated Th cells into the Th1 lineage by secreting cytokines such as IFN-γ and TNF-α and lead to more tissue damage [159].

In addition to its known properties, such as the ability to regenerate, MSC has some other exceptional properties that make them an excellent tool for use in medical science [160]. Today, MSCs are used to reduce various debilitating autoimmune disorders such as type 1 diabetes, SLE and MS [161]. One of the most essential benefits of MSC-based therapies is their ability to migrate preferentially to damaged tissues inflamed [162].

### Association of MSC–NK interactions with organ transplants

Recent studies have shown that in addition to T cells and B cells, NK cells play an essential role in innate and adaptive immune responses to the transplanted organ, and modulating their function may improve the transplant outcome [163]. NK cells can directly affect the transplanted organ through ADCC [164, 165] or by producing IFN-γ and increasing Th1 [163], leading to transplant rejection. Cyclosporine is used to prevent liver transplant (LT) rejection; as an immunosuppressant, it reduces graft rejection [166]. However, suppressing the immune system causes unavoidable complications such as opportunistic infections, viral recurrence, and LT patients' metabolic complications [167]. Also, it is recently indicated that NK cell function is not inhibited by conventional immunosuppressive drugs [168]. For this reason, researchers are searching for alternative methods to modulate the function of NK cells. Many researchers have turned their attention to MSCs because of their immunomodulatory properties [169]. In addition to being used in the therapy of various diseases, MSCs are also used in transplantation due to their role in preventing allograft rejection. They prevent transplant rejection by inhibiting the function and detrimental effects of NK cells on the transplanted organ [170]. New studies on MSC have shown that MSC transplantation is used as a significant factor in regulating the immune system to prevent transplant rejection and GVHD [171]. Researchers investigated the effect of MSC on survival or rejection of kidney transplantation by examining pre- and post-transplant donor-directed MLR. They found MSCs, in addition to inhibiting CD4+ and CD8+ T cell lymphocyte proliferation, could also prevent NK cell proliferation [172]. Huang et al. [173] stated that MSC can reduce the number of filtered NK cells in ischemic hind limbs. Therefore, it can be concluded that MSC can reduce NK cells in ischemic transplant organs to regulate the immune system against transplantation [168]. The suppressive effect of MSC on the immune system induced under inflammatory conditions such as IFN-γ-rich microenvironments [168]. Researchers have concluded that when pre-activated MSCs are used instead of naive MSCs in the transplant, extended the survival time of the transplant in pancreatic islet grafts [174]. It has also shown that the use of dexamethasone (135) and azathioprine (128) during MSC transplantation can improve the function of transplanted MSC in modulating the immune system.

## Conclusion

Over the past few years, many researchers have focused on the moderating effect of MSC on the immune system. The researchers showed MSC modulate the function of immune system cells, including NK cells. Some studies have shown that MSC suppresses NK cell function, while a few studies show that MSC enhances NK cell function. This discrepancy in the effect of MSC on NK cells could depend on co-culture conditions such as incubation time, MSC:NK cell ratio, pre-stimulated NK cell conditions. However, most studies indicate that MSC suppresses NK cell function. Today due to the effect of MSC on NK cell function, it has been considered as a therapeutic tool for the therapy of many diseases, including autoimmune diseases, and prevents transplant rejection and GVHD.

## Data Availability

Not applicable.

## Abbreviations

NK: Natural killer
MSC: Mesenchymal stem cell
GVHD: Graft-versus-host disease
PGE2: Prostaglandin E2
IDO: Indoleamine‐2,3‐dioxygenase
COX-2: Cyclooxygenase-2
BM: Bone marrow
NKR: NK receptor
KIR: Immunoglobulin-like receptor
ILT: Immunoglobulin-like transcript
IL-2R: Interleukin-2 receptor
BM-MSC: Bone marrow-derived mesenchymal stem cell
AT-MSC: Adipose tissue-derived mesenchymal stem cell
WJ-MSC: Wharton’s Jelly-derived mesenchymal stem cell
FSK-MSC: Foreskin-derived mesenchymal stem cell
LAIR-1: Leukocyte-associated Ig-like receptor-1
LAIR-2: Leukocyte-associated Ig-like receptor-2
FL-exo: Fetal liver MSC-derived exosomes
FL-MSC: Human fetal liver-derived mesenchymal stem cell
SOCS: Suppressor of cytokine signaling
IFN-γ: Interferon-γ
TNF-α: Tumor necrosis factor-alpha
IL-1β: Interleukin-1 β
HGF: Hepatocyte growth factor
HLA-G: Human leukocyte antigen G
